# Implementing quality management systems to close the AI translation gap and facilitate safe, ethical, and effective health AI solutions

**DOI:** 10.1038/s41746-023-00968-8

**Published:** 2023-11-25

**Authors:** Shauna M. Overgaard, Megan G. Graham, Tracey Brereton, Michael J. Pencina, John D. Halamka, David E. Vidal, Nicoleta J. Economou-Zavlanos

**Affiliations:** 1 Mayo Clinic, Rochester, MN USA; 2 Duke University, Durham, NC USA

**Keywords:** Health care, Medical research, Translational research

## Abstract

The integration of Quality Management System (QMS) principles into the life cycle of development, deployment, and utilization of machine learning (ML) and artificial intelligence (AI) technologies within healthcare settings holds the potential to close the AI translation gap by establishing a robust framework that accelerates the safe, ethical, and effective delivery of AI/ML in day-to-day patient care. Healthcare organizations (HCOs) can implement these principles effectively by embracing an enterprise QMS analogous to those in regulated industries. By establishing a QMS explicitly tailored to health AI technologies, HCOs can comply with evolving regulations and minimize redundancy and rework while aligning their internal governance practices with their steadfast commitment to scientific rigor and medical excellence.

## QMS as a framework for health AI

The advancements in healthcare software, encompassing artificial intelligence, machine learning (AI/ML), and Software as a Medical Device (SaMD), have brought about opportunities for transformative changes in clinical workflows and patient care to effectively meet patient and clinician needs. However, healthcare software exists within a complex regulatory and technical landscape^1^. The need for more readiness among healthcare organizations (HCOs) magnifies the disparity in translating research into effective predictive clinical decision support interventions. Without a collaborative enterprise approach, the intricate nature of this system delays the translation of AI solutions into clinical practice. Characterized by the continuous evolution and maturation of AI/ML capabilities, such as large language models (LLMs), this ecosystem escalates the demand for software-driven clinical solutions and a regulatory framework that must effectively adapt to govern the distinctive nature of in-house-built and procured software^2^. The growing engagement of HCOs in AI calls for alignment among diverse stakeholders, encompassing industry, academic institutions, and the medical community. This alignment should focus on harmonizing assurance standards for health AI technologies, but also practices and infrastructure to enable HCOs to develop and deploy AI solutions meeting rigorous medical-grade standards while ensuring accountability across all involved parties. While regulatory authorities, AI coalitions, medical device manufacturers, and the medical informatics community have acknowledged the current gap not only in common standards but also in the maturity of HCOs to develop and/or deploy health AI, a primary concern for HCOs remains unresolved: “How might our enterprise establish a coordinated, robust strategy that ensures the safe, effective, and ethically sound delivery of AI/ML in day-to-day patient care?”^3–7^.

We propose using the Quality Management System (QMS) framework to offer HCOs a consistent and adaptable structure to translate research-based health AI technology into clinical practice systematically and transparently. QMS is a structured framework that documents processes, procedures, and responsibilities to achieve quality policies and objectives. The QMS framework effectively manages evolving regulatory requirements, promotes continuous improvement, and ensures adherence to cutting-edge standards over the life cycle of the design, development, deployment, and maintenance of regulated healthcare software^8^. QMS’s are often certified to external standards (e.g., ISO 13485), thus demonstrating organizational commitment to quality, continuous improvement, and regulatory compliance. Aligning standards with risk-based approaches facilitates the least burdensome path for an HCO to meet regulatory requirements and maintain compliance^9^. Thus, the streamlined incorporation of these regulatory requirements into business processes via the QMS assures enduring safety, effectiveness, ethicality, regulatory compliance, and alignment with organizational and user needs as AI-enabled methodologies, such as LLMs, evolve^10^.

**Fig. 1:**
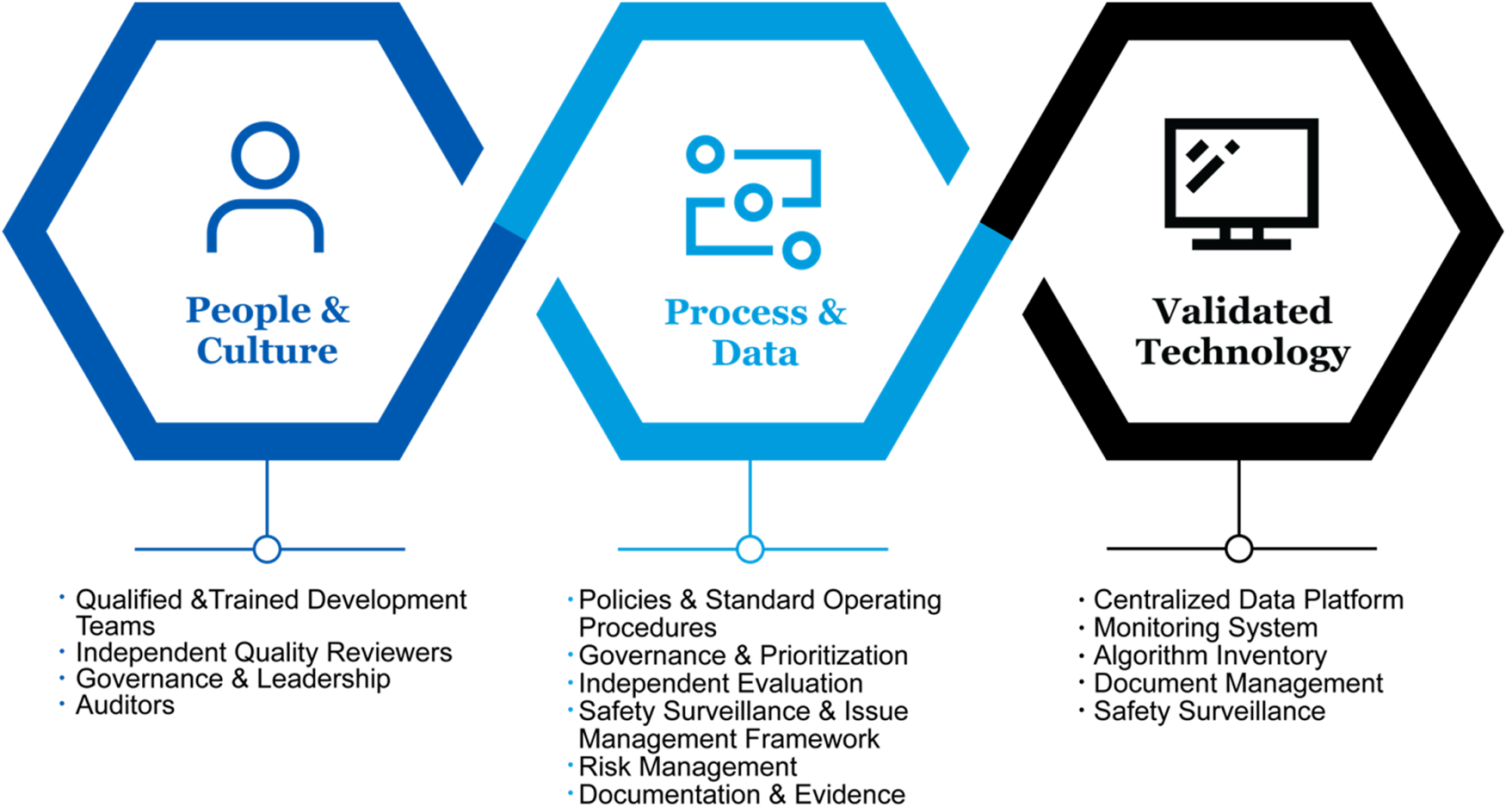
Primary components of a Quality Management System (QMS).

We aim to elucidate the primary components of a QMS (Fig. 1)^8,9,11^, namely People & Culture, Process & Data, and Validated Technology, as the impetus for HCO’s strategic efforts to integrate research rigor and clinical excellence into a cohesive system and close the AI translation gap.

## Establishing a proactive culture of quality

In HCOs, AI/ML technologies are often initiated as siloed research or quality improvement initiatives. However, when these AI technologies demonstrate potential for implementation in patient care, development teams may encounter substantial challenges and backtracking to meet the rigorous quality and regulatory requirements^12,13^. Similarly, HCO governance and leadership may possess a strong foundation in scientific rigor and clinical studies; however, without targeted qualifications and training, they may find themselves unprepared to offer institutional support, regulatory oversight, or mobilize teams toward interdisciplinary scientific validation of AI/ML–enabled technologies required for regulatory submissions and deployment of SaMD. Consequently, the unpreparedness of HCOs exacerbates the translation gap between research activities and the practical implementation of clinical solutions^14^. The absence of a systematic approach to ensuring the effectiveness of practices and perpetuating them throughout the organization can lead to operational inefficiencies or harm. Thus, HCOs must first contend with a culture shift when faced with quality control rigor inherent to industry-aligned software development and deployment, specifically design controls, version control, installation qualification, operational qualification, performance qualification, that primarily focuses on end-user acceptance testing and the product meeting its intended purpose (improving clinical outcomes or processes compared to the standard of care or the current state), and the traceability and auditability of proof records (Table 1).

**Table 1:** Common QMS and Medical Device Terminology.

Term	Definition
End-User Acceptance Testing	Testing that demonstrates that the product satisfies end-user defined requirements^12^.
Corrective and Preventive Action (CAPA)	Changes to products and/or the quality management system to eliminate the cause of or prevent the occurrence of issues that affect the quality of products or processes^9^.
Design Controls	Interrelated set of practices and procedures that are incorporated into the design and development process, i.e., a system of checks and balances^8,12^.
Design Verification and Validation	Testing or evaluation activities that provide objective evidence that the product satisfies its intended purpose (or use) and specifications^9^.
Installation Qualification	Testing or evaluation activities that demonstrate the software is installed correctly in its intended environment^12^.
Intended Purpose (Intended Use)	The objective intent and general purpose of a product^29^.
Operational Qualification	Testing or evaluation activities that demonstrate the software functions as intended^12^.
Performance Qualification	Testing or evaluation activities that demonstrate the software satisfies user and business requirements^12^.
Records	Documents or other artifacts that demonstrate that a process was completed^8^.
Software Development Life Cycle (SDLC)	A framework of processes, activities, and tasks phases to design, develop, and maintain software^30^.
Monitoring and Surveillance	Tracking and analyzing the performance of and changes to products after deployment to identify quality or safety issues that may necessitate corrective or preventive action^9^.
Version Control	Activity related to software configuration management intended to track and manage changes to software. Version control is used during all stages of the software development life cycle to prevent mix-ups^8,30^.

Consider that even in cases where a regulatory submission is not within the scope, it remains imperative to adhere to practices encompassing ethical and quality principles. Examples of such principles identified by the Coalition for Health AI and the National Institute for Standards and Technology (NIST) include effectiveness, safety, fairness, equity, accountability, transparency, privacy, and security^3,7,15–20^. It is also feasible that the AI/ML technology could transition from a non-regulated state to a regulated one due to updated regulations or an expanded scope. In that case, a proactive approach to streamlining the conversion from a non-regulatory to a regulatory standard should address the delicate balance of meeting baseline requirements while maintaining a least-burdensome transition to regulatory compliance.

As utilized by the FDA for regulating SaMD, a proactive culture of quality recognizes the same practices familiar to research scientists well-versed in informatics, translational science, and AI/ML framework development. For example, the FDA has published good machine learning practices (GMLP)^21^ that enumerate its expectations across the entire AI/ML life cycle grounded in emerging AI/ML science. The FDA’s regulatory framework allows for a stepwise product realization approach that HCOs can follow to augment this culture shift. This stepwise approach implements ethical and quality principles by design into the AI product lifecycle, fostering downstream compliance while allowing development teams to innovate and continuously improve and refine their products. Using this approach allows for freedom to iterate at early research stages. As the product evolves, the team is prepared for the next stage, where prospectively planned development, risk management, and industry-standard design controls are initiated. At this stage, the model becomes a product, incorporating all the software and functionality needed for the model to work as intended in its clinical setting. QMS procedures outline practices, and the records generated during this stage create the level of evidence expected by industry and regulators^22,23^. HCOs may either maintain dedicated quality teams responsible for conducting testing or employ alternative structures designed to carry out independent reviews and audits.

**Fig. 2:**
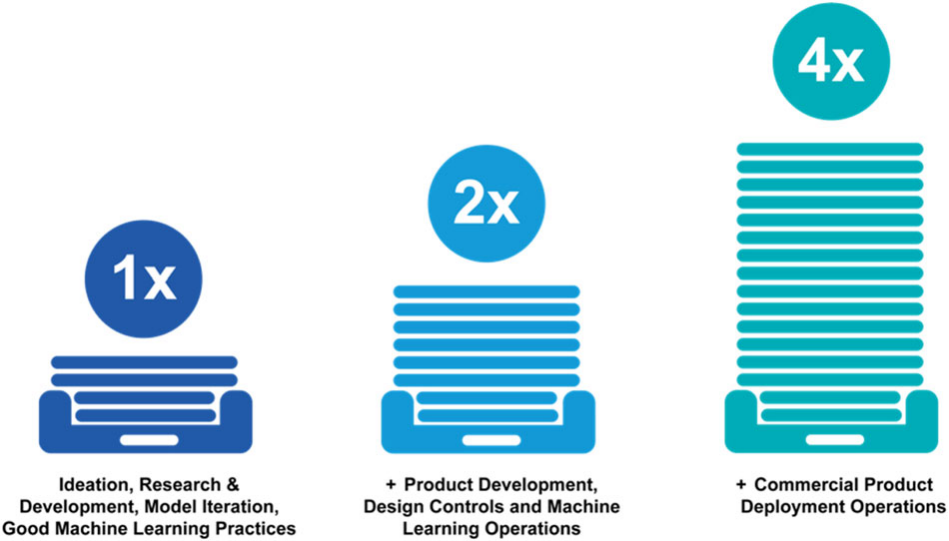
Staged process for applying increasing regulatory rigor throughout product realization.

Upon deployment, QMS rigor increases again to account for standardized post-deployment monitoring and change management practices embedded in QMS procedures (Fig. 2). By increasing formal QMS consistency as the AI/ML gets closer to clinical deployment, the QMS can minimize disruption to current research practices and embolden HCO scientists with a clear pathway as they continue to prove their software safe, effective, and ethical for clinical deployment.

## Establishing risk-based design, development, and monitoring

The medical device industry has utilized a risk-based infrastructure for years to support a least burdensome approach to designing, developing, and deploying healthcare technologies^9,24^. This approach systematically enables HCOs to proactively focus resources on key areas of concern, such as safety, equity, and data privacy, to prevent errors and malfunctions and promote a culture of accountability and continuous improvement.

**Fig. 3:**
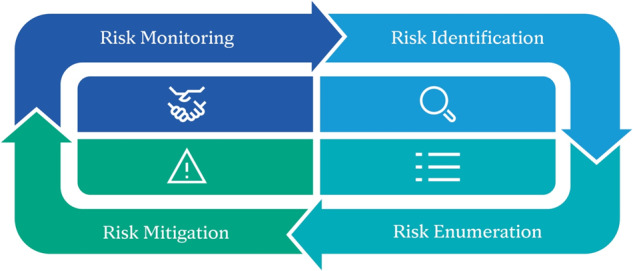
Example QMS risk management plan and risk assessment phases. Risks are identified, assessed and analyzed, mitigated and controlled, and continuously monitored. Reporting is performed at pre-defined intervals.

Risk-based practices have been extended to healthcare AI/ML in not only the medical device domain, such as with AAMI’s Technical Information Report 34971^25^, but more broadly in emerging frameworks such as the NIST AI Risk Management Framework^3^, the Whitehouse Blueprint for an AI Bill of Rights^5^, the Coalition for Health AI Blueprint for Trustworthy AI Implementation Guidance and Assurance for Healthcare^26^, and the Health AI Partnership Key Decision Points^27,28^. Risk management is grounded in the intended use and informed by a prospective risk management plan. It follows the process of identification, enumeration, mitigation, and monitoring (Fig. 3) to analyze and classify potential sources of harm (known as hazards) caused by the healthcare software or its impact on the clinical workflow. As the healthcare software is designed and developed, features or attributes that reduce or minimize the risk (known as mitigations) are included in the product design; for example, incorporating features that improve the user experience or providing user training or documentation to clarify how the software should or should not be used. As risks and potential issues are anticipated for the health software’s implementation, a risk management plan is put in place, a document articulating how safety, bias, and other anticipated risks will be identified and resolved. Risks continue to be monitored, reported, and reviewed after the software is deployed to ensure the software remains safe for use. Systematic feedback, monitoring, and corrective & preventive action (CAPA) frameworks are key to identifying and triaging issues, escalating issues to relevant accountable departments of the organization depending on their severity, performing root-cause analysis, and continuously controlling risks and improving the AI technology.

Risk-based practices formalized and implemented within a QMS will systematically identify risks associated with an AI solution, document mitigation strategies, and offer a framework for objective testing and auditing of individual technology components. Further, such technologies can be informed by AI/ML and software life cycle best practices to address common issues within phases of the AI lifecycle. This allows for capturing performance metrics across various levels of rigor and data transparency in requirements, version, and design controls. These insights from initial testing can then support the calibration and maintenance of AI solutions during deployment, guided by a multidisciplinary governance system to proactively mitigate future risks^26^. Moreover, establishing a change management plan and access controls can eliminate business continuity risks, providing transparency into responsible parties and outlining the risks of any given change. Back-up (downtime) processes are in place in the event that risk cannot be managed, and the technology needs to be turned off. Effectively, a risk-based approach ensures the proper rigor and controls are in place at the right time throughout the product life cycle.

## Establishing a compliance-facilitating infrastructure

The regulations for healthcare software are evolving. Software may or may not be regulated based on its intended use or by changes to regulatory agency enforcement. A QMS that facilitates compliance with applicable legal and regulatory requirements enables HCOs to design, implement, and deploy healthcare software to clinical practice while minimizing overall operational risk. A QMS fosters compliance to internal (e.g., institutional review board) and external (e.g., federal, and local regulatory) bodies by standardizing multi-faceted stakeholder responsibilities with its governance, allowing auditability and traceability through the appropriate evidence and documentation, maintaining an inventory of AI technologies developed and deployed, and hosting infrastructure that will allow document management and monitoring within the deployment platform.

A QMS involves establishing policies and standard operating procedures that outline the process for governance and prioritization, development, independent evaluation, maintenance and monitoring, issue reporting and safety surveillance. Procedures outline the roles and responsibilities of stakeholders such as design and testing responsibilities of the champion stakeholder representing the end-users in the product development process. Procedures should also articulate training and/or qualification requirements for the stakeholders participating in AI technology development teams as safety and other risks can be eliminated with stakeholder education. Procedures also outline the systems and communication channels available to the community impacted by the deployed algorithmic tools ensuring their compliance. Communication in a regulated QMS is bidirectional, where issues, safety surveillance and outcome data are gathered via real-time monitoring and tightly integrated with the risk management and patient safety operations of a given healthcare system to determine the behavior and impact on patients and their healthcare delivery.

Establishing an innovation infrastructure that facilitates compliance requires governance and leadership support to create a communicated mandate that all algorithmic tool-related activities impacting patient health comply with quality and ethical standards. For example, the governing body may have direct integration with existing IRB processes to ensure ethical conduct. With proper governance, algorithm inventory, and transparency, HCOs can begin to implement tools, testing, and monitoring capabilities into their QMS to reduce the burden and achieve safe, effective, ethical ML/AI at scale. Implementing QMS involves formal documentation encompassing quality, ethical principles, and processes, ensuring transparency and traceability to regulatory requirements.

## Conclusion

HCOs can utilize a QMS framework to accelerate the translation of AI from research to clinical practice. A proactive quality culture, risk-based framework for design, development, monitoring, and compliance-oriented infrastructure enables continuous ethical review, ensuring the effectiveness, safety, and equity of AI/ML technologies and meet regulatory requirements. Implementing a QMS requires adaptability, customization, and interdisciplinary collaboration, fostering awareness, education, and organizational growth. Drawing on regulatory precedents and incorporating insights from expert stakeholders, the QMS framework enables HCOs to prioritize patient needs and foster trust in adopting innovative AI technologies, including those enabled by LLMs.
